# Determinants of Walking among Middle-Aged and Older Overweight and Obese Adults: Sociodemographic, Health, and Built Environmental Factors

**DOI:** 10.1155/2017/9565430

**Published:** 2017-07-04

**Authors:** Samuel N. Forjuoh, Marcia G. Ory, Jaewoong Won, Samuel D. Towne, Suojin Wang, Chanam Lee

**Affiliations:** ^1^Department of Family & Community Medicine, Baylor Scott & White Health, College of Medicine, Texas A&M Health Science Center, Temple, TX, USA; ^2^Department of Health Promotion & Community Health Sciences, School of Public Health, Texas A&M University, College Station, TX, USA; ^3^Department of Landscape Architecture & Urban Planning, College of Architecture, Texas A&M University, College Station, TX, USA; ^4^Department of Statistics, College of Science, Texas A&M University, College Station, TX, USA

## Abstract

**Background:**

This study examined the association between selected sociodemographic, health, and built environmental factors and walking behaviors of middle-aged and older overweight/obese adults.

**Methods:**

Subjective data were obtained from surveys administered to community-dwelling overweight/obese adults aged ≥50 years residing in four Texas cities from October 2013 to June 2014, along with objective data on neighborhood walkability (Walk Score™). Multivariate logistic regression identified factors predicting the odds of walking the recommended ≥150 minutes per week for any purpose.

**Results:**

Of 253 participants, the majority were non-Hispanic white (81.8%), married (74.5%), and male (53.4%) and reported an annual income of ≥$50,000 (65.5%). Approximately, half were employed (49.6%) or had at least a college degree (51.6%). Walking the recommended ≥150 minutes per week for any purpose (*n* = 57, 22.5%) was significantly associated with having at least a college degree (OR = 5.55, 95% CI = 1.79–17.25), having no difficulty walking a quarter of a mile (OR = 5.18, 95% CI = 1.30–20.83), and being unemployed (OR = 3.25, 95% CI = 1.18–8.93) as well as perceived presence of sidewalks/protected walkways (OR = 3.56, 95% CI = 1.10–11.50) and perceived absence of distracted drivers in the neighborhood (OR = 4.08, 95% CI = 1.47–11.36).

**Conclusion:**

Addressing neighborhood conditions related to distracted drivers and pedestrian infrastructure may promote walking among middle-aged and older overweight/obese individuals.

## 1. Introduction

The global public health burden of obesity is well established as is the influence of physical activity on the health and well-being of individuals who are overweight or obese [1, 2]. In the United States, obesity is a major public health problem for all ages, but particularly for middle-aged and older adults who do not seem to engage in adequate physical activity. This is a timely issue given projections that those aged 65 years or older are expected to nearly double between 2012 and 2050 [3]. In fact, obesity is higher among middle-aged and older adults than among their younger counterparts [4]. Approximately two-thirds of the adult population in the United States is overweight and nearly one-third is obese [4, 5], yet most do not get adequate physical activity [6].

The federal 2008 Physical Activity Guidelines for Americans recommend adults to avoid inactivity and have at least 150 minutes of moderate-intensive aerobic activity weekly and muscle-strengthening activities on two or more days every week. For older adults who cannot do 150 minutes of moderate-intensive aerobic activity due to chronic conditions, the recommendation is for them to be physically active as their abilities and conditions allow [7]. However, most middle-aged and older adults do not meet this guideline. In 2014, the percentage of adults aged 45–54 years and 55–64 years who met the federal 2008 Physical Activity Guidelines for Americans for both aerobic activity and muscle-strengthening exercise was 19.3% and 16.1%, respectively, compared to 25.1% and 31.1% for those aged 25–44 years and 18–24 years, respectively. The corresponding rates for those aged 65–74 years and 75 years or older were 14.4% and 7.9%, respectively [8]. Physical activity in old age is particularly important for disease prevention, health maintenance, quality of life, overall independence, and fall prevention [7, 9, 10]. Preventing or delaying chronic diseases is a salient issue as nearly nine in 10 older adults have at least one chronic condition, with nearly three in four having at least two chronic conditions [11]. Thus, identifying factors that may lessen the burden of chronic disease as individuals age is especially timely with this rapidly aging population [3].

Although there are numerous ways to be physically active, walking is one of the most popular and frequently recommended forms of physical activity, especially for middle-aged and older adults who may have difficulty with certain forms of rigorous physical activity and in line with the current recommendation of ≥150 minutes of exercise per week (as opposed to specifying 30 minutes of moderate-intensity exercise five days per week) for older adults [12, 13]. In fact, walking or jogging was rated the highest among older adults' favorite activities in a large nationally representative sample of older adults [14].

Walking is important for preventing obesity as well as promoting better health and reducing healthcare costs for those who are already obese [15, 16]. Cognizant of the physical activity guidelines by the physicians in our setting [17], walking was cited as the most common activity recommended by family physicians for their obese patients, along with bicycling and swimming [18]. Regular physical activity such as walking can prevent or ameliorate the effects of many chronic conditions that afflict middle-aged and older adults, lower their quality of life, and contribute to the leading causes of death. Therefore, promoting walking as a form of physical activity in middle-aged and older adults can directly benefit them through improved quality of life and reduced burden of chronic conditions. This is especially true for middle-aged and older adults who are overweight or obese.

While prior studies have uncovered predictors of walking among older adults, including the barriers and motivators [19, 20], less is known about the predictors of walking among middle-aged and older adults who are overweight or obese. The predictors for the latter population could be different given the added morbidity or chronic medical condition to their aging condition. In an exploratory study, Lee et al. [21] addressed barriers and motivators to walking among overweight and obese adults, but the sample included all adults aged 18 years or older and the focus was on age variations in barriers or motivators with little mention of health predictors of walking among this population. They, however, concluded that overweight and obese adults may experience considerable modifiable environmental barriers to walking.

This study examined factors associated with the walking behaviors of middle-aged and older overweight and obese adults, focusing on the influence of selected sociodemographic, health, and built environmental factors. In line with the behavioral-environment health model [21], it is important to understand which multilevel factors are most correlated with reported walking as a form of physical activity. Additionally important is having both subjective and objective measures of the built environment.

## 2. Methods

### 2.1. Study Design, Setting, and Participants

A cross-sectional survey was conducted from October 2013 to June 2014 in a large university-affiliated, integrated multispecialty healthcare system associated with a 220,000-member Health Maintenance Organization in four central Texas cities (Temple, Killeen, College Station, and Bryan). These cities included peri-urban environments where urban and rural features coexist within cities, which can exhibit diverse neighborhood contexts with a range of street and development patterns from car-dependent to fairly walkable environments. The survey used a standardized, online, and paper questionnaire that included open-ended and multiple choice questions. Study participants were community-dwelling overweight or obese adults aged ≥50 years residing in one of the four cities and seen by a family physician in a primary care clinic in the previous three years who reported some level of walking as a form of physical activity. Objective data were also collected using Walk Score to capture walkability (i.e., accessibility to destinations) of the survey respondent's residential neighborhood. The study protocol was reviewed and approved by the Institutional Review Boards of Baylor Scott & White Health and the Texas A&M University.

### 2.2. Recruitment Procedure and Data Collection

Details of the research methods, including the procedures for recruitment and data collection, have been published elsewhere [19, 22, 23]. In brief, participants were recruited via electronic medical records (EMRs) that were utilized to conduct initial patient screening by age and geographic residence. Primary care providers were then provided with a list generated from the EMRs to identify potentially eligible patients from a sampling list, from which 7,543 recruitment letters were sent out. The sample was restricted to those who (a) had no difficulty reading, writing, and speaking English; (b) were not terminally ill; and (c) did not live in a nursing home or assisted-living community. In order to ensure patients' capability of walking as a form of physical activity, screening questions on the ability to walk in their neighborhood without major difficulty or for a quarter of a mile (approximately 2-3 city blocks) or for about 5–10 minutes were included at the front of the survey instrument.

The link to the online survey was sent to participants who opted for the online version (69.5%) via provided email addresses, while paper surveys were mailed to those who opted for the paper version (30.5%) to their provided addresses. Designed to take approximately 20 minutes to complete, the survey included questions on sociodemographic, health, and built environmental factors potentially related to walking. Previously validated or tested instruments including the Behavioral Risk Factor Surveillance System developed by the Centers for Disease Control and Prevention [24], Starting-The-Conversation developed by the University of North Carolina Prevention Partners [25], the International Physical Activity Questionnaire [26], and the Neighborhood Environment Walkability Scale [27] informed the survey. Each participant was offered a $10 gift card at the completion of the survey.

### 2.3. Study Measures

The primary study outcome measure was walking the recommended ≥150 minutes per week for any purpose. This measure of walking behavior was obtained by combining two survey items that estimated walking days for any purpose in a typical week and walking minutes for any purpose in each day. The outcome variable was dichotomized into (a) walking ≥150 minutes per week for any purpose (as a proxy for meeting the federal 2008 Physical Activity Guidelines for Americans) and (b) walking <150 minutes per week for any purpose (as a proxy for not meeting the guidelines for physical activity).

We used Walk Score as one of our predictor variables to measure walkability of each participant's residential environment [28–30]. Walk Score is calculated based on an accessibility-based measure to various walkable destinations from home, providing an objective and disaggregated estimate of walkability around each participant's residence. Walk Score can be used to categorize areas that are walkable (Walk Score ≥ 50) versus those that are car-dependent (Walk Score < 50) [28]. Neighborhood perception, captured from the survey, was conceptualized with several questions related to the built environments that focused on (1) neighborhood safety from traffic, for example, “There is so much traffic along nearby streets that it makes it difficult or unpleasant to walk in my neighborhood”; (2) neighborhood safety from crime, for example, “My neighborhood streets are well lit at night”; (3) neighborhood safety from physical injury risks, for example, “There are many broken sidewalks in my neighborhood”; (4) behavioral factors in neighborhood safety, for example, “There are many distracted drivers in my neighborhood (e.g., on the cell phone while driving)”; (5) social aspects of the neighborhood, for example, “People in my neighborhood know each other”; and (6) attractiveness of the neighborhood, for example, “There are many attractive buildings, homes or gardens to see in my neighborhood.”

Other predictor variables were also captured from the survey and included anthropometric (i.e., being overweight or obese), social interaction (e.g., someone to walk with, dog in household), and demographic characteristics (e.g., age, gender, education) as well as health (e.g., difficulty walking for a quarter of a mile and health condition) variables.

### 2.4. Statistical Analyses

Descriptive statistics were used to characterize participants' anthropometric and socio-demographic data as well as their health conditions, walking, and built environmental behaviors. Pearson chi-square tests were then used to assess differences between participants who reported walking versus not walking the recommended ≥150 minutes per week. Finally, multivariate logistic regression was used to predict walking the recommended ≥150 minutes per week for any purpose using the selected sociodemographic, health, and built environmental factors. Those variables that showed statistical significance in the bivariate tests and were clinically sensible and plausible were added to the multivariate model. Adjusted odds ratios and 95% confidence limits of the final multivariate model are reported. All analyses were conducted in 2016 using Stata 13 (StataCorp, College Station, TX). Statistical significance was set at the *P* < 0.05 level.

## 3. Results

### 3.1. Survey Response and Walking Behaviors

Of the 496 patients who participated in the survey (adjusted response rate, 6.8%), 102 participants were excluded for not meeting the study eligibility criteria (e.g., did not live in our study areas, lived in assisted facilities, and aged <50 years) or screening criteria (e.g., did not walk) or completing the survey. Of the remaining 394 participants, 141 were excluded for not being overweight or obese. Analyses for this study were therefore based on the remaining 253 participants who were overweight or obese adults, of whom 57 (22.5%) reported walking the recommended ≥150 minutes per week for any purpose.

**Figure 1:**
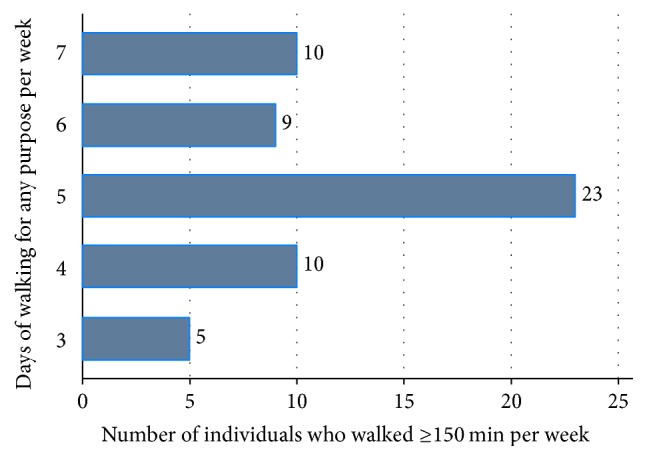
Number of days of walking for any purpose by middle-aged and older overweight and obese individuals who walked ≥150 minutes per week.

**Figure 2:**
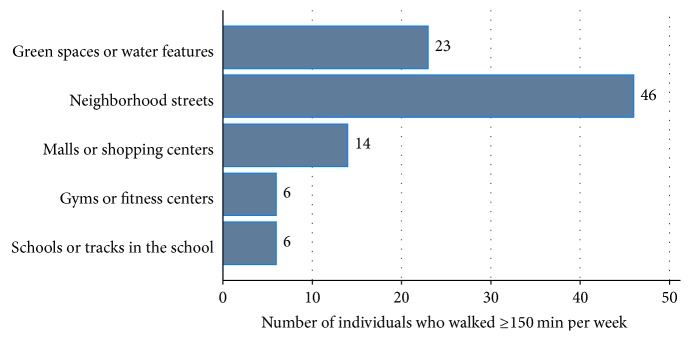
Places for walking for any purpose by middle-aged and older overweight and obese individuals who walked ≥150 minutes per week.

Of the 57 who reported walking the recommended ≥150 minutes per week for any purpose, most (40.4%, *n* = 23) reported walking 5 days per week, 17.5% (*n* = 10) reported walking 7 days per week, and another 17.5% (*n* = 10) reported walking 4 days per week, while 15.8% (*n* = 9) reported walking 6 days per week and 8.8% (*n* = 5) reported walking 3 days per week (Figure 1). The most popularly used places for walking were the neighborhood streets, green places with water features, and malls or shopping centers (Figure 2).

### 3.2. Anthropometric, Demographic, and Health Characteristics

Of the total sample who were overweight or obese adults (*n* = 253), the majority were overweight (55.7%), non-Hispanic white (81.8%), married (74.5%), and male (53.4%) and reported an annual household income of ≥$50,000 (65.4%). Approximately, half were aged ≥65 years (48.2%), employed (49.6%), or had at least a college degree (51.6%). They were generally healthy, with 51.0% reporting very good to excellent health and 35.6% reporting good health. Overall, 23.8% reported having a little or some difficulty walking for a quarter of a mile. Nearly half of them reported receiving a primary care provider recommendation to be more physically active (Table 1).

### 3.3. Social Support and Built Environmental Characteristics

Table 2 summarizes the bivariate social support and built environmental characteristics of the overweight or obese participants. Few respondents reported having caregiving responsibilities for elders (15.1%), while a substantial number reported having a dog in the household (41.9%), and almost half of them reported having someone to walk with (49.0%). The vast majority (91.3%) resided in less walkable or car-dependent areas (Walk Score < 50) than walkable areas (Walk Score ≥ 50). The mean and median Walk Scores were 20.4 and 17.0, respectively, with a range from 0 to 72. However, no significant differences were seen between those who walked the recommended ≥150 minutes per week for any purpose and those who did not in terms of these social support variables.

Of the built environmental factors related to neighborhood safety from traffic, one showed a significant difference between those overweight or obese adults who walked the recommended ≥150 minutes per week for any purpose and those who did not. Among those overweight or obese adults who walked the recommended ≥150 minutes per week for any purpose, significantly more participants strongly agreed/agreed that there are crosswalks and pedestrian signals to help walkers cross busy streets in the neighborhood than those who strongly disagreed/disagreed (26.4% versus 18.2%, *P* = 0.01). Similarly, one variable among the built environmental factors related to neighborhood safety from physical injury risk showed a significant difference between the two groups. Significantly more overweight or obese participants strongly agreed/agreed that they were worried about falling when walking in the neighborhood than those who strongly disagreed/disagreed (25.4% versus 11.4%, *P* = 0.045). None of the other built environmental variables related to neighborhood safety from crime and behavioral risk factors showed statistically significant differences.

### 3.4. Factors Associated with Walking the Recommended ≥150 Minutes per Week for any Purpose among Overweight or Obese Adults in Multivariate Model

The strongest determinants of overweight or obese participants walking the recommended ≥150 minutes per week for any purpose in a multivariate model (Table 3) were related to respondent's educational levels and favorable neighborhood perceptions. Compared to those with a high school diploma or some college, participants with a college degree or higher were more than five times as likely to walk the recommended ≥150 minutes per week for any purpose (OR = 5.55, 95% CI = 1.79–17.25). Walking the recommended ≥150 minutes per week for any purpose was significantly associated with participants who strongly disagreed/disagreed that there were many distracted drivers (e.g., on the cell phone while driving) in their neighborhood (OR = 4.08, 95% CI = 1.47–11.36) and those who strongly agreed/agreed that there are sidewalks or protected walkways (e.g., walking trails) in their neighborhood (OR = 3.56, 95% CI = 1.10–11.50). Those employed were significantly less likely to walk the recommended ≥150 minutes per week compared to those unemployed (OR = 0.31, 95% CI = 0.11–0.85) as were those who reported some difficulty walking for a quarter of a mile (OR = 0.19, 95% CI = 0.05–0.77).

Nearly threefold more participants strongly disagreed/disagreed that drivers do not yield to pedestrians or bicyclists in the neighborhood than those who strongly agreed/agreed, meaning that most felt that drivers do yield. The disagreement with “Drivers do not yield to pedestrians or bicyclists in my neighborhood” predicted a borderline association with extra walking (*P* = 0.087). As would be expected, there was a positive relationship between extra walking and having a dog in the household, although the trend was also only borderline in strength (*P* = 0.098). Being overweight or obese and health condition as well as other social interaction factors such as having someone to walk with and care-giver responsibilities for elders as well as Walk Score were not significantly associated with the odds of walking the recommended ≥150 minutes per week for any purpose among overweight or obese adults.

Counter-intuitively, walking the recommended ≥150 minutes per week for any purpose was significantly associated with participants who strongly agreed/agreed that most drivers exceeded the posted speed limits while driving, strongly disagreed/disagreed that walkers and bikers on the streets can be easily seen, and strongly agreed/agreed that there is so much traffic along nearby streets (Table 3).

## 4. Discussion

In this cross-sectional study of community-dwelling overweight and obese adults aged 50 years or older in central Texas, 22.5% reported walking the recommended ≥150 minutes per week for any purpose and three personal variables, including having at least a college degree, not having any difficulty walking for a quarter of a mile, and not being employed, were identified as significant personal level determinants of walking the recommended ≥150 minutes per week for any purpose after controlling for other covariates. Unfortunately, no comparable state or national data on the percentage of overweight and obese adults in a comparable age group who walked ≥150 minutes per week could be found.

From the neighborhood built environmental variables, strong disagreement with the perception that there are many distracted drivers (e.g., on the cell phone while driving) in one's neighborhood was found to be positively associated with walking the recommended ≥150 minutes per week for any purpose. Further, strong agreement that there are sidewalks or protected walkways (e.g., trails) in one's neighborhood was found to be associated with the increased odd of walking ≥150 minutes per week for any purpose. Thus, the relative importance of having sidewalks and protected walkways in one's neighborhood for walking can serve as a target for environmental interventions that focus on either maintaining or building such structures. It is also possible that those who walk more are more likely to know the pedestrian infrastructure in the neighborhood, such as sidewalks and walkways. Therefore, programs and campaigns to increase awareness of pedestrian infrastructure available in the neighborhood, as well as policies aimed to reduce distracted drivers, may also require further attention as components of future walking intervention programs. To the best of our knowledge, this is the first study to assess determinants of walking among a population of overweight and obese middle-aged and older adults. However, these factors are the same as found for older adults of all weight status, including normal weight [19].

Features in the built environment may be more favorable for certain segments of the population such as those who are more educated, less healthy, unemployed, or retired [31]. In the current study, walking the recommended ≥150 minutes per week for any purpose was found to be associated with individuals with at least a college degree and those unemployed possibly including those who are retired. Higher education can enhance an individual's knowledge about health promotion, including the need for more physical activity such as walking [32, 33] even for those who may be overweight or obese as found in this study. Thus, those seeking to promote physical activity campaigns and other interventions can use the results of this study to set targets and to tailor marketing materials. Similarly, physicians seeking to encourage patients to be more active may also use this information to target and tailor encouragement for physical activity to particularly at-risk patients (e.g., those with lower educational levels). Also, being unemployed or retired may be associated with the needed time to engage in more physical activity such as walking [34].

Safety from traffic and behavioral risks such as that found in this study, along with safety from crime and injury risks, are among the foremost factors for promoting walking [35]. Exposure of middle-aged or older adults to increased risks of being hit by a distracted driver due to lack of sidewalks or protected walkways may deter them from walking. In fact, older adults bear disproportionately high rates of fatality from traffic crashes, accounting for 18% of all pedestrian deaths, although they account for only 13% of the total population [36]. Neighborhoods without distracted drivers and with ample safety features such as sidewalks or protected walkways will definitely promote walking even among middle-aged and older overweight and obese adults.

Despite the fact that the majority of the participants in this study resided in less walkable areas, one in five reported walking the recommended ≥150 minutes per week for any purpose. This finding is encouraging and may imply that enhancing the walkability of the neighborhood will make more individuals walk in their neighborhood. Prior studies have shown associations between higher neighborhood walkability and decreased prevalence of overweight and obesity, possibly via increased physical activity such as walking [37, 38].

Several seemingly counter-intuitive associations were found with walking the recommended ≥150 minutes per week for any purpose. These included strong agreement that most drivers exceeded the posted speed limits while driving and that there was so much traffic along nearby streets making it difficult or unpleasant to walk in one's neighborhood as well as strong disagreement that walkers and bikers on the neighborhood streets can be easily seen by people from their homes and were rather unusual. However, these findings are likely due to the increased awareness of or sensitivity to those neighborhood conditions influencing pedestrians among those who walk more. For example, those who walk a lot (meeting the recommended ≥150 minutes) are more likely to witness and consider inadequate driver behaviors, lack of visual surveillance, and heavy traffic to be problematic. The observation of so much traffic can be balanced with some observed positive, albeit insignificant, driver behavior of yielding to pedestrians or bicyclists. This observation may indicate that heavy traffic is associated with neighborhood walking, but safe driving behaviors should be encouraged. Although designing the roadway to reduce traffic is very important, driver behavior towards pedestrians may be more important for promoting neighborhood walking.

Our study had some limitations that must be taken into consideration in the interpretation of the findings. First, the limitation of our study to the selected four sites reduces the generalizability of the findings as was the exclusion of non-English speaking subjects which may have missed others, particularly Hispanics, who are a large subset with documented overweight and obesity problems. Second, our study had a very low response rate, further impacting the generalizability of study findings. However, this is typical of studies that depend on EMRs for subject recruitment [21, 39], and the actual refusal rate once a patient was identified was rather very low (0.15%, 11 out of 7,543). Further, our intent was to explore the associations between different correlates and walking behaviors in an understudied population rather than to conduct a larger more national epidemiological study. Third, the survey item for walking behavior we used was “how many days in a typical week do you walk in your neighborhood.” We did not thoroughly define respondents' neighborhood boundary, and the definition might have been different across respondents. Finally, the cross-sectional design of the study precludes determination of causality.

## 5. Conclusions

In conclusion, we found that neighborhood perceptions and built environmental characteristics appear to be important determinants of the walking behaviors of middle-aged and older overweight and obese individuals as presented previously [40] and to be presented in an upcoming conference [41]. Enhancing the neighborhood environments, especially those related to pedestrian walkways, driver behaviors, visual surveillance, and traffic conditions, has the potential to promote walking among these individuals as well as the general population. Such enhancement may include providing walking trails and beautiful neighborhoods conducive for walking. However, these neighborhood environment enhancements ought to take cognizance of the needs of middle-aged and older individuals who may be overweight or obese.

## Figures and Tables

**Table 1:** Anthropometric, demographic, and health characteristics of study participants.

Variable	Total participants (*N* = 253)		Walked ≥150 min per week for any purpose (*n* = 57)		Walked <150 min per week for any purpose (*n* = 196)		*P* value
	FREQ^a^	%	FREQ^a^	% ^b^	FREQ^a^	%^b^	
Weight status							0.94
Overweight	141	55.7	32	22.7	109	77.3	
Obese	112	44.3	25	22.3	87	77.7	
Age group (years)							0.65
50–64	131	51.8	28	21.4	103	78.6	
≥65	122	48.2	29	23.8	93	76.2	
Gender							0.63
Male	135	53.4	32	23.7	103	76.3	
Female	118	46.6	25	21.2	93	78.8	
Race/ethnicity							0.53
Other	46	18.2	12	26.1	34	73.9	
Non-Hispanic white	206	81.8	45	21.8	161	78.2	
Marital status							0.22
Divorced/widowed	64	25.5	11	17.2	53	82.8	
Married/living with a partner	187	74.5	46	24.6	182	75.4	
Education							**0.012** ^*∗*^
High school or some college	118	48.4	18	15.2	100	84.8	
College graduate or graduate	126	51.6	36	28.6	90	71.4	
Employment							0.198
Not employed	126	50.4	33	26.2	93	73.8	
Employed	124	49.6	24	19.3	100	80.7	
Annual household income							0.070
<$50,000	85	34.6	14	16.5	71	83.5	
≥$50,000	161	65.4	43	26.7	118	73.3	
Difficulty walking for a quarter of a mile							**0.002** ^*∗∗*^
Not at all difficult	192	76.2	52	27.1	140	72.9	
Only a little/somewhat	60	23.8	5	8.3	55	91.7	
Health condition							0.37
Poor to fair	34	13.4	5	14.7	29	85.3	
Good	90	35.6	19	21.1	71	78.9	
Very good to excellent	129	51.0	33	25.6	96	74.4	
Received PCP recommendation to be more physically active							0.177
No	130	51.8	34	26.1	96	73.9	
Yes	121	48.2	23	19.0	98	81.0	

Boldface indicates statistical significance (^*∗*^*P* < 0.05; ^*∗∗*^*P* < 0.01). ^a^May not add to total due to missing data. ^b^Row %. PCP = primary care provider.

**Table 2:** Social and built environmental characteristics of study participants' neighborhood.

Variable	Total participants (*N* = 253)		Walked ≥150 min per week for any purpose (*n* = 57)		Walked <150 min per week for any purpose (*n* = 196)		*P* value
	FREQ^a^	%	FREQ^a^	%^b^	FREQ^a^	%^b^	
Someone to walk with							0.75
No	129	51.0	28	21.7	101	78.3	
Yes	124	49.0	29	23.4	95	76.6	
Have caregiving responsibilities for elders							0.80
No	214	84.9	49	22.9	165	77.1	
Yes	38	15.1	8	21.0	30	79.0	
Dog in household							0.21
No	147	58.1	29	19.7	118	80.3	
Yes	106	41.9	28	26.4	78	73.6	
Walk Score (WS)							0.61
WS < 50 (less walkable)	231	91.3	53	22.9	178	77.1	
WS ≥ 50 (walkable)	22	8.7	4	18.2	18	81.8	
^†^There is so much traffic along nearby streets that it makes it difficult to walk							0.35
Strongly agree/agree	73	28.7	19	26.4	53	73.6	
Strongly disagree/disagree	181	71.3	37	20.9	140	79.1	
^†^Most drivers exceed the posted speed limits while driving in my neighborhood							0.94
Strongly agree/agree	120	47.8	27	22.5	93	77.5	
Strongly disagree/disagree	131	52.2	30	22.9	101	77.1	
^†^There are sidewalks or protected walkways (e.g., trails) in my neighborhood							0.12
Strongly disagree/disagree	110	44.0	20	18.2	90	81.8	
Strongly agree/agree	140	56.0	37	26.4	103	73.6	
^†^There are crosswalks and pedestrian signals to help walkers cross busy streets							**0.01** ^*∗*^
Strongly disagree/disagree	163	65.5	28	17.2	135	82.8	
Strongly agree/agree	86	34.5	27	31.4	59	68.6	
^‡^My neighborhood streets are well lit at night							0.44
Strongly disagree/disagree	109	43.1	22	20.2	87	79.8	
Strongly agree/agree	144	56.9	35	24.3	109	75.7	
^‡^Walkers and bikers on the streets can be easily seen by people from their homes							0.98
Strongly disagree/disagree	162	65.3	36	22.2	126	77.8	
Strongly agree/agree	86	34.7	19	22.1	67	77.9	
^‡^I see and speak to other people when I am walking in my neighborhood							0.07
Strongly disagree/disagree	42	16.7	5	11.9	37	88.1	
Strongly agree/agree	210	83.3	52	24.8	158	75.2	
^*¥*^There are many broken sidewalks in my neighborhood							0.57
Strongly agree/agree	63	25.2	16	25.4	47	74.6	
Strongly disagree/disagree	187	74.8	41	21.9	146	78.1	
^*¥*^I am worried about falling when I walk in my neighborhood							**0.045** ^*∗*^
Strongly agree/agree	205	82.3	52	25.4	153	74.6	
Strongly disagree/disagree	44	17.7	5	11.4	39	88.6	
^*¥*^My neighbors could be counted on to help in case of need							0.16
Strongly disagree/disagree	42	16.7	6	14.3	36	85.7	
Strongly agree/agree	210	83.3	51	24.3	159	75.7	
^§^There are many distracted drivers in my neighborhood (e.g., on phone while driving)							0.199
Strongly agree/agree	156	61.7	31	19.9	125	80.1	
Strongly disagree/disagree	97	38.3	26	26.8	71	73.2	
^§^Drivers do not yield to pedestrians or bicyclists in my neighborhood							0.26
Strongly agree/agree	83	33.6	15	18.1	68	81.9	
Strongly disagree/disagree	164	66.6	40	24.4	124	75.6	
^§^There is drug dealing in my neighborhood							0.43
Strongly agree/agree	211	85.8	49	23.2	162	76.8	
Strongly disagree/disagree	35	14.2	6	17.1	29	82.7	

Boldface indicates statistical significance (^*∗*^*P* < 0.05). ^a^May not add to total due to missing data. ^a^Row %. ^†^Traffic safety. ^‡^Crime safety. ^*¥*^Physical injury risk safety. ^§^Behavioral risk safety.

**Table 3:** Adjusted ORs and 95% CI of walking the recommended ≥150 minutes per week for any purpose.

Variable	OR	95% CI	*P* value
Education			
High school or some college	1.00	—	
College graduate or graduate school	5.55	1.79–17.25	**0.003** ^*∗∗*^
Difficulty walking for a quarter of a mile			
Not at all difficult	1.00	—	
Only a little or somewhat difficult	0.19	0.05–0.77	**0.020** ^*∗*^
There are many distracted drivers in the neighborhood			
Strongly agree/agree	1.00	—	
Strongly disagree/disagree	4.08	1.47–11.36	**0.007** ^*∗∗*^
There are sidewalks or protected walkways in neighborhood			
Strongly disagree/disagree	1.00	—	
Strongly agree/agree	3.56	1.10–11.50	**0.034** ^*∗*^
Drivers do not yield to pedestrians or bicyclists			
Strongly agree/agree	1.00	—	
Strongly disagree/disagree	2.67	0.87–8.22	0.087
^¶^Most drivers exceed the posted speed limits while driving			
Strongly disagree/disagree	1.00		
Strongly agree/agree	2.85	1.10–7.41	**0.031** ^*∗*^
^¶^Walkers and bikers on the streets can be easily seen			
Strongly agree/agree	1.00		
Strongly disagree/disagree	4.00	1.17–13.70	**0.027** ^*∗*^
^¶^There is so much traffic along nearby streets			
Strongly disagree/disagree	1.00		
Strongly agree/agree	4.33	1.47–12.66	**0.008** ^*∗∗*^
Employment			
Not employed	1.00	—	
Employed	0.31	0.11–0.85	**0.022** ^*∗*^
Annual household income			
<$50,000	1.00	—	
≥$50,000	1.55	0.47–5.20	0.474
Age group (years)			
50–64	1.00	—	
≥65	0.87	0.30–2.51	0.791
Race/ethnicity			
Other	1.00	—	
Non-Hispanic white	0.35	0.10–1.21	0.096
Marital status			
Divorced, widowed, separated, or never married	1.00	—	
Married or living with a partner	0.73	0.20–2.63	0.625
Gender			
Male	1.00	—	
Female	0.78	0.30–2.06	0.621
Weight status			
Overweight	1.00	—	
Obese	1.30	0.52–3.26	0.570
Health condition			
Poor to fair	1.00	—	
Good	3.13	0.47–20.67	0.237
Very good to excellent	6.87	0.96–49.33	0.055
Someone to walk with			
No	1.00	—	
Yes	0.51	0.19–1.38	0.183
Dog in household			
No	1.00	—	
Yes	2.07	0.87–4.90	0.098
Has caregiving responsibilities for elders			
No	1.00	—	
Yes	0.64	0.17–2.33	0.495
Walk Score			
Less walkable	1.00	—	
Walkable	1.71	0.40–7.27	0.467

*N* = 209. Likelihood Ratio Chi^2^ = 53.33 (*P* = 0.014). Pseudo *R*^2^ = 0.245. Boldface indicates statistical significance (^*∗*^*P* < 0.05; ^*∗∗*^*P* < 0.01). ^¶^Counter-intuitive association.
